# Poverty and health-related quality of life: a cross-sectional study in rural China

**DOI:** 10.1186/s12955-020-01409-w

**Published:** 2020-05-26

**Authors:** Zhong Li, Liang Zhang

**Affiliations:** 1 School of Medicine and Health Management, Tongji Medical College, Huazhong University of Science and Technology, No.13 Hangkong Road, Wuhan, 430030 Hubei China; 2 Research Center for Rural Health Service, Key Research Institute of Humanities & Social Sciences of Hubei Provincial Department of Education, Wuhan, 430030 Hubei China

**Keywords:** Poverty, Health-related quality of life, EQ-5D-3 L, Heterogeneous effect, Rural China

## Abstract

**Background:**

The association between poverty and health has been widely assessed. However, whether the association between poverty and health-related quality of life (HRQOL) holds among different groups is unknown. This study aimed to 1) assess the association between poverty and HRQOL among rural residents in China and 2) examine whether the association holds among different populations, thereby supporting policy-making and implementation.

**Methods:**

A multistage, stratified, random household survey was conducted with self-administered questionnaires. Matched samples were generated by the censored exact matching method to reduce selection bias between the poverty and comparison groups. We applied Tobit and ordinal logit regression models to evaluate the association between poverty and HRQOL measured by the EQ-5D-3 L among different groups.

**Results:**

The health utility score of the poverty group was 6.1% lower than that of comparison group (95% CI = − 0.085, − 0.037), with anxiety/depression being most common (95% CI = 1.220, 1.791). The association between poverty and HRQOL was significantly stronger among residents from central China, males, people who were middle-aged, elderly, highly educated, married, or widowed, those living far from healthcare facilities, and those without chronic disease. Male and highly educated subjects reported worse mobility, self-care, usual activities, pain/discomfort and anxiety/depression dimensions than the other respondents. Middle-aged (95% CI = 1.692, 2.851) and married respondents (95% CI = 1.692, 2.509) and respondents with chronic diseases (95% CI = 1.770, 2.849) were most affected in the anxiety/depression.

**Conclusions:**

The HRQOL of individuals living in poverty is lower than that of the general population, and the mental health dimension is most affected by poverty among respondents who are middle-aged or married and respondents with chronic diseases. The identification of populations that are more affected by poverty is critical to improve their HRQOL. Various associations have indicated the need for integrated policies and specific decision-making.

## Background

Health creates wealth and is one of the key contributors to Sustainable Development Goals, especially for poverty reduction and the promotion of health and well-being [1]. The world still faces considerable challenges in terms of poverty issues [2]. A previous study noted the importance of health and economic well-being by using new measures of poverty-free life expectancy [3]. Given the slowing of global economic growth, governments must maximise the benefits of economic input during the process of poverty elimination [4].

In China, a study revealed that the heavy burden of out-of-pocket (OOP) payments has become a poverty trap for poor individuals [5]. In India, 4.1% of the population was in a state of hidden poverty caused by high medical expenses and an undeveloped health insurance system in 2011/2012 [6]. Wagstaff et al. [7] demonstrated that OOP expenditure causes approximately 100 million individuals to fall into poverty annually. Remarkable health disparities are mainly driven by income [8]; that is, health and poverty are inextricably associated [9–11]. In the United States, the emergence of the health poverty trap has continuously widened health inequalities within populations of different income levels [9]. Chetty et al. [10] also found that high income is associated with considerable longevity, with a gap of approximately 15 years for men and 10 years for women between the poorest 1% and the richest 1% of individuals. Poverty and income inequality also induce poor mental health via multiple material and psychosocial channels [12–14]. In China, the incidence of poverty decreased from 97.5% in 1978 to 1.7% in 2018 [15]. However, the social security system does not provide sufficient support for vulnerable populations with high health needs, which may result in these populations falling into or returning to poverty [16, 17]. In 2016, the Central Chinese Government established a target for poverty reduction: “By 2020, lifting all individual living in poverty out of the poverty under the local criteria in rural China” [18]. Therefore, investigating the association between poverty and health will facilitate effective healthcare resource allocation for poor and other disadvantaged populations.

The mechanism underlying the impact of poverty on physical and mental health within different populations has been widely examined, including the following potential channels: 1) lifestyle, living status [19], consumption [20] and connectedness with the external environment [21, 22]; 2) health investments [23, 24]; and 3) increased financial stress [19]. However, whether the association between poverty and HRQOL holds among different groups has rarely been investigated, thereby leaving a research gap for future investigation.

## HRQOL measurement

As a common indicator to assess health system performance based on respondents’ preferences [25–27], the EQ-5D-3 L instrument plays a vital role in many population health surveys limited to health administration data [28, 29], especially in developing countries [30, 31]. The descriptive system of the EQ-5D-3 L includes five dimensions: 1) mobility, 2) self-care, 3) usual activities, 4) pain/discomfort and 5) anxiety/depression. Each dimension comprises three response levels: 1) none, 2) some and 3) extreme/unable to. The EQ Visual Analogue Scale assesses respondents’ self-rated health with a 20 cm vertical ruler, with the endpoint ranging from 0 (the worst health one can imagine) to 100 (the best health one can imagine). HRQOL is calculated by adding the scores corresponding to each item response, that is, by converting the EQ-5D states into a health utility.

In China, numerous studies have focused on HRQOL among different populations, including migrant females [32] and children, using various scales [33]. A systematic review suggested that many factors influence HRQOL, including age, gender, comorbidities, and rural/urban status [34]. However, whether the association between poverty and HRQOL holds among different groups remains unknown. Therefore, this study aimed to 1) assess the association between poverty and HRQOL among rural residents in China and 2) examine whether the association holds among different populations, thereby supporting policy-making and implementation. As the first study to investigate the association between poverty and HRQOL with the coarsened exact matching (CEM) method, this paper contributes to research on the poverty–health relationship. It also supports adjustment and implementation strategies related to poverty elimination in China and other developing countries with similar settings.

## Methods

### Study design and data collection

First, socioeconomic development and geographic distribution and suggestions from experts were assessed. Two counties (DY, Dangyang in Central China; SN, Sinan in Western China) were purposively selected [35]. Second, a multistage stratified random household survey was conducted with face-to-face interviews. Given that the design effect of a well-designed, multistage stratified study is between 2 and 2.5 [36–40], we set the design effect (DEFF) as 2.5 in this study.

1 $$ {n}_i={u}_a^2\cdot p\cdot \left(1-p\right)/{\delta}^2 $$

2 $$ {N}_i={n}_i\cdot DEFF $$

Equation (1) is used to calculate the sample size: *δ* is the allowable error for the significance level of *α* = 0.05, and *p* is the prevalence of chronic diseases among the Chinese population according to the 2013 National Health Service Survey [41]. Equation (2) is used to calculate the sample size of a multistage sample. DEFF is the effect of the complex survey design on sampling variance measured as the ratio of the sampling variance under the complex design and the sampling variance obtained from a simple random sample of the same sample size [42]. Therefore, the sample size in each study site was 3584 residents. Third, given that in China, one family has an average of 2.9 members [41], 1235 families had to be surveyed. To control the overall budget and to keep the design effect as low as possible, 30 primary sample units were selected in DY and SN. Five townships were randomly selected according to the distance to the county hospitals. In each township, six villages were randomly selected according to the distance to the township hospitals (5*6 = 30). Hence, 42 families were interviewed in each village. Self-administered questionnaires on socioeconomic status, living status, presence/absence of poverty, health insurance, health status measured by the EQ-5D-3 L instrument, healthcare utilisation, and the presence/absence of chronic diseases were completed by the respondents. A total of 2735 families with 7293 individuals were interviewed face to face from July 2018 to August 2018.

In China, the population in poverty is currently identified based on the average annual income (National Line of Poverty: 4000 Chinese Yuan in 2020), limited access to education and healthcare services and a low standard of living. Hence, the poverty group was identified according to the enrolment list of the local government. The EQ-5D-3 L instrument is designed for residents aged ≥15 years old, and 1134 respondents aged < 15 years old were excluded. Furthermore, 48 individuals who failed to complete the questionnaires were excluded. Finally, 6111 respondents comprised the original database in this study, including 791 and 5368 respondents in the poverty and comparison groups, respectively. This study was approved by the Ethics Committee of Tongji Medical College, Huazhong University of Science and Technology (IORG No: IORG0003571).

### Statistical analysis

In this study, the generic EQ-5D-3 L utility instrument for the Chinese population, which ranges from 0.149 to 1, was used to measure HRQOL [43]. First, the frequency and mean value of all variables were calculated. Bivariate analysis was performed to assess the differences between the poverty and comparison groups. Second, the CEM method was used to overcome the imbalance between the poverty and comparison groups. Third, Tobit models and ordinal logit models were used to investigate the association between poverty and HRQOL. Although the EQ-5D-3 L includes only five items and is not perfectly comparable with other comprehensive instruments on mental disorders, one study revealed that somatic and psychological symptoms are related to the EQ-5D-3 L [44]. Thus, the EQ-5D-3 L can aid in screening mental disorders [45]. We applied the ordinary logit model to estimate the association between the dimensions of poverty and mobility, self-care, usual activities, pain/discomfort and anxiety/depression [21]. In addition, the heterogeneous effects were estimated to identify whether the association held among different groups. The significance level was set at *p* < 0.05.

### CEM method

In the traditional regression model, potential confounding factors may result in selection bias on the overall effect estimation. In this study, the CEM method proposed by Lacus et al. [46, 47] was applied to maintain good balance between different groups and generate accurate estimation results. This method has been widely adopted because of the following advantages compared with propensity score matching: 1) common empirical support is not needed, and 2) the original sample is generated during the matching process. This method included three main steps: 1) coarsening each variable with recoding to group and assigning the incomparable values the same value, 2) using exact matching and 3) removing coarsened data and reserving the final matched data. Finally, the variable generated was used to weigh and equalise the number of observations within different groups. Multivariate imbalance measuring L1 was used to check the balance between the multivariate histogram and assess the matching performance. For any given set of groups, if two distributions are completely separated, then L1 = 1; if the distributions exactly coincide, then L1 = 0. Therefore, L1 ranged from 0 to 1, its substantial reduction indicated good matching performance. For example, if L1 = 0.6, then 40% of the areas under the two histograms overlap [46, 47].

### Covariate variables for matching

According to a previous literature review, three levels of factors were applied as covariates for matching: 1) individual level: age, gender, marital status, education level, enrolment in social or commercial health insurance and presence/absence of chronic diseases; 2) family level: household size and distance to the nearest healthcare facilities; and 3) region, which was used as an ecological variable to characterise the local context in each county [48].

## Results

### Demographic information of the participants

As shown in Table 1, among the 6111 respondents, 49.12% were male. The poverty incidence in SN was higher than that in DY (19.36% vs. 6.03%, *p* < 0.001). The differences between the two groups in terms of age (*p* = 0.003), marital status (*p* < 0.001) and level of educational attainment (*p* < 0.001) were statistically significant. The incidence of poverty also increased as the distance to the nearest healthcare facilities increased (*p* < 0.001). The poverty group showed a higher rate of being enrolled in social health insurance (*p* = 0.008) and suffering from chronic diseases (*p* < 0.001) than the comparison group. The differences between the two groups in terms of gender (*p* = 0.706), household size (*p* = 0.624) and enrolment in social health insurance (*p* = 0.577) were not statistically significant. Middle-aged residents showed the highest incidence of falling into poverty, followed by the elderly. The level of educational attainment was also negatively correlated with the poverty incidence (*p* < 0.001).

**Table 1:** Demographic characteristic for the poverty and comparison group

Variables	Categories	Overall (*N* = 6111)	Comparison (*N* = 5328)	Poverty (*N* = 783)	Chi(2)	*P*
Region	DY	3002 (49.12)	2821 (93.97)	181 (6.03)	243.08	< 0.001
	SN	3109 (50.88)	2507 (80.64)	602 (19.36)		
Gender	Male	2998 (49.12)	2609 (87.02)	389 (12.98)	0.14	0.706
	Female	3106 (50.88)	2713 (87.35)	393 (12.65)		
Age	15–44	1545 (25.28)	1345 (87.06)	200 (12.94)	11.61	0.003
	45–59	2860 (46.8)	2532 (82.24)	328 (17.76)		
	> 60	1706 (27.92)	1451 (85.05)	255 (14.95)		
Marital status	Single	588 (9.66)	467 (79.42)	121 (20.58)	43.32	< 0.001
	Married	4882 (80.23)	4324 (88.57)	558 (11.43)		
	Widowed	551 (9.06)	467 (84.75)	84 (15.25)		
Distance ^a^	< 2 km	4968 (81.36)	4373 (88.02)	595 (11.98)	17.94	< 0.001
	1–2 km	745 (12.2)	631 (84.70)	114 (15.30)		
	> 3 km	393 (6.44)	321 (81.68)	72 (18.32)		
Household size^b^	median (p25, p75)	3 (2,4)	3 (2,4)	3 (2,4)	−0.49	0.623
Education level	≤PS	3184 (53.16)	2670 (83.86)	514 (16.14)	68.00	< 0.001
	JS	1939 (32.37)	1761 (90.82)	178 (9.18)		
	≥SS	867 (14.47)	792 (91.35)	75 (8.65)		
SHI	Yes	6051 (99.51)	5274 (87.16)	777 (12.84)	1.10	0.577
	No	40 (0.66)	35 (87.5)	5 (12.5)		
CHI	Yes	498 (8.17)	453 (90.96)	45 (9.04)	6.99	0.008
	No	5596 (91.83)	4859 (86.83)	737 (13.17)		
Number of NCDs	0	3428 (56.25)	3039 (57.19)	389 (49.81)	15.12	< 0.001
	≥1	2666 (43.75)	2274 (42.81)	392 (50.19)		

Note: N (row %) was reported. *DY* Dangyang, *SN* Sinan, *PS* primary school, *JS* junior school, *SS* senior school, *CHI* Commercial health insurance, *SHI* Social health insurance, *NCD* Chronic Non-Communicable Diseases. ^a^Distance to the nearest healthcare facilities, including the primary care centers or pharmacy. ^b^For the distribution of household size is not normal, Median (p25, p75) was reported and Kruskal-Wallis test was used

### Matching performance

As shown in Table 2, the multivariate L1 statistics between the poverty and comparison groups decreased close to 0 after matching, thereby indicating good matching performance and increasing the comparability of the two groups. The univariate L1 value also indicated that the two groups matched well. A new database consisting of 671 respondents in the poverty group and 3344 respondents in the comparison group was generated to estimate the effects.

**Table 2:** The L1 measure of imbalance before and after the coarsened exact matching

Variables	before matching L1(mean)	after matching L1(mean)
Region	0.30265 (0.30265)	1.6e-15 (4e-15)
Gender	0.00177 (−0.00177)	1.2e-15 (0)
Age	0.05425 (0.0583)	2.9e-15 (−6.7e-15)
Marital status	0.08921 (−0.02041)	1.1e-15 (−8.4e-15)
Distance ^a^	0.0538 (0.08406)	9.4e-16 (2.2e-16)
Household size	0.04367 (0.08231)	2.4e-15 (8.7e-15)
Education level	0.16496 (−0.22206)	1.5e-15 (−2e-15)
SHI	0.02789 (−0.02789)	8.6e-16 (−1.7e-16)
CHI	0.07514 (−0.07514)	1.1e-15 (5.3e-15)
Multivariate L1	0.50114	0.00797
N	6111	4015

Note: *CHI* Commercial health insurance, *SHI* Social health insurance; ^a^Distance to the nearest healthcare facilities

### Descriptive analysis of the five dimensions and health utility scores

As shown in Table 3, 18.57, 12.16, 19.28, 33.56 and 21.85% of the respondents reported problems in mobility, self-care, usual activities, pain/discomfort and anxiety/depression, respectively. The median value of the health utility value was 0.783. After matching, the median value was 0.862 in the comparison group, which was higher than that of the poverty group (*p* < 0.001). Comparison between the two groups showed that the respondents in the poverty group exhibited a higher rate of reporting worse HRQOL in the five dimensions, especially in terms of anxiety/depression (*p* < 0.001).

**Table 3:** Self-reported health status in the five dimensions and EQ-5D index

Dimension	Overall (*N* = 6111)	Comparison (*N* = 3344) ^a^	Poverty (*N* = 671) ^a^	Chi(2)	*P*
		N (%)			
**Mobility**					
I have no problems in walking out	4976 (81.43)	2485 (74.31)	470 (70.04)	7.32	0.026
I have some problems in walking out	1084 (17.74)	833 (24.91)	191 (28.46)		
I am confined to bed	51 (0.83)	26 (0.78)	10 (1.49)		
**Self-Care**					
I have no problems with self-care	5368 (87.84)	2804 (83.85)	537 (80.03)	6.49	0.039
I have some problems in washing or dressing myself	645 (10.55)	480 (14.35)	116 (17.29)		
I am unable to wash or dress myself	98 (1.6)	60 (1.79)	18 (2.68)		
**Usual Activity**					
I have no problems with performing my usual activities	4933 (80.72)	2501 (74.79)	463 (69.00)	11.5	0.003
I have some problems with performing my usual activities	1046 (17.12)	766 (22.91)	183 (27.27)		
I am unable to perform my usual activities	132 (2.16)	77 (2.3)	25 (3.72)		
**Pain/Discomfort**					
I have no pain or discomfort	3877 (63.44)	1884 (56.34)	337 (50.22)	10.37	0.006
I have moderate pain or discomfort	2120 (34.69)	1386 (41.45)	311 (46.35)		
I have extreme pain of discomfort	114 (1.87)	74 (2.21)	23 (3.43)		
**Anxiety/Depression**					
I am not anxious or depressed	4776 (78.15)	2340 (69.98)	424 (63.19)	21.76	< 0.001
I am moderately anxious or depressed	1290 (21.11)	982 (29.37)	233 (34.72)		
I am extremely anxious or depressed	45 (0.74)	22 (0.66)	14 (2.09)		
**EQ-5D index, median (p25, p75)**^b^	1 (0.783, 1)	1 (0.862, 1)	0.869 (0.690, 1)	92.95	< 0.001

Note: ^a^ after matching. ^b^For the distribution of household size is not normal, Median (p25, p75) was reported and Kruskal-Wallis test was used

### Association between poverty and HRQOL

The results of regression models using the matched sample are demonstrated in Table 4. The health utility scores of the poverty group were 6.1% lower than those of the comparison group (95% CI = − 0.085, − 0.037). The poverty group showed a higher likelihood of reporting worse HRQOL for mobility (95% CI = 1.088, 1.713), self-care (95% CI = 1.171, 1.742), usual activities (95% CI = 1.178, 1.721), pain/discomfort (95% CI = 1.220, 1.791) and anxiety/depression (95% CI =1.075, 1.614) than the comparison group. The anxiety/depression dimension was most affected (OR = 1.478).

**Table 4:** Results of the Tobit regression and ordinal multi-nominal regression model

Dependent variables	Coefficient (95% CI)	LR chi2(17)	Pseudo R^**2**^
EQ-5D index	− 0.061 (− 0.085, − 0.037) ^a^	1356.80^a^	0.3469
Mobility	1.318 (1.075,1.614) ^b^	806.75^a^	0.1652
Self-Care	1.363 (1.088,1.713) ^b^	546.91^a^	0.1348
Usual Activity	1.428 (1.171,1.742) ^a^	764.21^a^	0.1468
Pain/Discomfort	1.423 (1.178,1.721) ^a^	1195.20^a^	0.1930
Anxiety/Depression	1.478 (1.220,1.791) ^a^	727.45 ^a^	0.1384

Note: Number of observations, 4015; The covariate variables are region, gender, age, marital status, distance, household size, education, social health insurance, commercial health insurance, chronic disease or not; ^a^ and ^b^ represent statistical significance at the 0.1, 1 and 5% levels, respectively

### Heterogeneous effects

As shown in Table 5, the point estimates for the health utility scores were larger for DY residents (95% CI = − 0.148, − 0.038), male respondents (95% CI = − 0.132, − 0.053) and young subjects (95% CI = − 0.142, − 0.073). Respondents who received an education of junior school (95% CI = − 0.173, − 0.104), senior school or above (95% CI = − 0.334, − 0.073) and those who were married (95% CI = − 0.128, − 0.074) or widowed (95% CI = − 0.175, − 0.038) were more affected than their counterparts. Respondents living far away from healthcare facilities were also the most affected by poverty (95% CI = − 0.220, − 0.077). The association between poverty and HRQOL was stronger for respondents without chronic disease (95% CI = − 0.153, − 0.068). Regarding the five dimensions, respondents from DY were more likely to report problems with self-care (95% CI =1.43, 2.504) and usual activities (95% CI = 1.603, 3.702). Male subjects and respondents with a high level of educational attainment reported worse HRQOL. The elderly population (> 60) also tended to report worse HRQOL for self-care (95% CI = 0.884, 2.408). The young groups (15–44) exhibited a high probability of suffering from worse HRQOL in terms of mobility (95% CI = 1.290, 5.806), usual activities (95% CI = 1.141, 4.622) and pain/discomfort (95% CI = 1.538, 4.305). The middle-aged groups mostly suffered from anxiety/depression (95% CI = 1.692, 2.851). Widowed respondents tended to suffer in terms of mobility (95% CI = 1.027, 2.962), usual activity (95% CI = 1.234, 3.560) and pain/discomfort (95% CI = 1.446, 4.567). Married respondents tended to report worse HRQOL in terms of self-care (95% CI = 0.841, 2.136) and anxiety/depression (95% CI =1.692, 2.509). Residents living far from healthcare facilities reported worse HRQOL than those living near those services in terms of the five dimensions, with the exception of pain/discomfort. For respondents with chronic diseases, anxiety/depression was the dimension that was most affected (95% CI = 1.770, 2.849).

**Table 5:** Heterogeneous effect of poverty on the HRQOL and the five dimensions

Variables	Categories	NO	EQ-5D index	Mobility^a^	Self-Care^a^	Usual Activity^a^	Pain/Discomfort^a^	Anxiety/Depression^a^
Region	DY	1554	−0.093 (− 0.148, − 0.038) ^d^	1.541 (0.950, 2.504)	2.547 (1.43, 2.504) ^d^	2.437 (1.603, 3.702) ^d^	1.362 (0.918, 2.019)	1.272 (0.670, 2.413)
	SN	2422	−0.053 (− 0.081, − 0.026) ^e^	1.284 (1.019, 1.617) ^f^	1.193 (0.438, 1.617)	1.257 (0.998, 1.582)	1.440 (1.154, 1.796) ^e^	1.494 (1.215, 1.840) ^d^
Gender	Male	1947	−0.092 (− 0.132, − 0.053) ^d^	1.481 (1.100, 1.993) ^e^	1.590 (0.794, 1.993) ^e^	1.582 (1.185, 2.112) ^e^	1.526 (1.164, 2.003) ^e^	1.759 (1.329, 2.327) ^d^
	Female	2037	−0.037 (− 0.069,0.690) ^f^	1.192 (0.899, 1.582)	1.184 (0.484, 1.582)	1.296 (0.984, 1.709)	1.328 (1.017, 1.733) ^f^	1.276 (0.982, 1.658)
Age	15–44	1018	−0.120 (− 0.195, − 0.046) ^e^	2.737 (1.290, 5.806) ^e^	1.348 (1.26, 5.806)	2.297 (1.141, 4.622) ^f^	2.575 (1.538, 4.305) ^d^	1.587 (0.992, 2.542)
	45–59	2134	−0.107 (− 0.142, − 0.073) ^d^	1.576 (1.171, 2.121) ^e^	1.743 (0.906, 2.121) ^e^	1.898 (1.424, 2.529) ^d^	1.675 (1.296, 2.166) ^d^	2.196 (1.692, 2.851) ^d^
	> 60	1458	−0.090 (− 0.130, − 0.049) ^d^	1.805 (1.353, 2.408) ^d^	1.793 (0.884, 2.408) ^d^	1.748 (1.317, 2.323) ^d^	1.474 (1.096, 1.979) ^e^	1.913 (1.426, 2.570) ^d^
Education level	≤ PS	2781	−0.081 (− 0.109, − 0.052) ^d^	1.537 (1.232, 1.919) ^d^	1.563 (0.694, 1.919) ^d^	1.675 (1.347, 2.083) ^d^	1.505 (1.218, 1.857) ^d^	1.847 (1.503, 2.272) ^d^
	JS	2527	−0.138 (− 0.173, − 0.104) ^d^	2.019 (1.503, 2.710) ^d^	2.181 (1.126, 2.710) ^d^	2.300 (1.729, 3.055) ^d^	1.930 (1.502, 2.481) ^d^	2.583 (1.993, 3.346) ^d^
	≥ SS	528	−0.204 (− 0.334, − 0.073) ^d^	5.238 (1.275, 21.49) ^f^	^c^	12.12 (3.167, 46.38) ^d^	2.593 (0.865, 7.767)	2.247 (0.904, 5.584)
Marital Status	Single	360	0.093 (−0.215,0.029)	2.669 (0.796, 8.944)	0.489 (0.099, 2.416)	1.587 (0.477, 5.275)	2.003 (0.740, 5.419)	1.246 (0.529, 2.927)
	Married	3842	−0.101 (−0.128, − 0.074) ^d^	1.717 (1.381, 2.136) ^d^	1.814 (0.841, 2.136) ^d^	1.823 (1.474, 2.254) ^d^	1.592 (1.306, 1.940)	2.060 (1.692, 2.509) ^d^
	Widowed	408	−0.107 (− 0.175, − 0.038) ^d^	1.745 (1.027, 2.962) ^f^	1.676 (1.072, 2.962)	2.098 (1.234, 3.560) ^e^	2.570 (1.446, 4.567) ^e^	1.809 (1.065, 3.074) ^f^
Distance ^b^	< 2 km	4000	−0.062 (− 0.127, − 0.071) ^d^	1.313 (1.075, 1.603) ^d^	1.362 (0.534, 1.603) ^d^	1.430 (1.174, 1.740) ^d^	1.411 (1.171, 1.704) ^d^	1.429 (1.190, 1.714) ^d^
	2–3 km	397	−0.149 (− 0.220, − 0.077) ^e^	2.457 (1.306, 4.618) ^e^	2.403 (1.582, 4.618) ^f^	2.787 (1.532, 5.068) ^e^	1.685 (0.968, 2.932)	1.902 (1.070, 3.380) ^f^
	> 3 km	223	−0.067 (− 0.145, − 0.012)	2.437 (1.168, 5.083) ^f^	2.349 (1.674, 5.083) ^f^	1.478 (0.735, 2.968)	1.146 (0.534, 2.464)	1.698 (0.838, 3.441) ^f^
No of NCDs	0	2488	−0.111 (− 0.153, − 0.068) ^d^	1.573 (1.121, 2.205) ^e^	1.995 (1.097, 2.205) ^e^	1.888 (1.362, 2.622) ^d^	1.755 (1.336, 2.307) ^d^	1.635 (1.237, 2.161) ^e^
	≥1	2122	−0.100 (− 0.131, − 0.069) ^d^	1.833 (1.430, 2.349) ^d^	1.643 (0.759, 2.349) ^d^	1.811 (1.424, 2.300) ^d^	1.611 (1.262, 2.056) ^d^	2.245 (1.770, 2.849) ^d^

Note: ^a^ordinal logit regression models were applied; ^b^Distance to the nearest healthcare facilities, including the healthcare centers or pharmacy: ^c^Not concave; The covariate variables are region, gender, age, marital status, distance, household size, education, social health insurance, commercial health insurance, chronic disease or not; *NO* number of observation, *DY* Dangyang, *SN* Sinan, *PS* primary school, *JS* junior school, *SS* senior school, *NCD* Chronic Non-Communicable Diseases; ^d^, ^e^ and ^f^ represent statistical significance at the 0.1, 1 and 5% levels, respectively; Coefficient (95% CI) was reported

## Discussion

To the best of our knowledge, this study is the first to identify the association between poverty and HRQOL in rural China. First, we compared the basic characteristics between the two groups. Second, we used the CEM method to remove selection bias between the two groups, thereby estimating the association between poverty and HRQOL against the background of poverty reduction in China. Third, the heterogeneous effects were estimated to identify the most vulnerable populations.

### Distribution of the five dimensions and health utility scores

Respondents from western rural China showed higher poverty rates than those from central rural China. Middle-aged population and widowed and single respondents were also associated with an increased likelihood of poverty. The incidence of poverty was also associated with healthcare accessibility, educational attainment and the number of chronic diseases. The most frequently reported problem was pain/discomfort, which was observed in almost half of the respondents; this proportion was much higher than the national average in 2013 [41].

### Association between poverty and HRQOL

The poverty group reported low health utility scores and worse performance on the five dimensions, indicating high disease burden. Poverty affected the anxiety/depression status of respondents in the majority of the dimensions. These findings were consistent with previous studies on the negative association between mental health and poverty [22, 49]. Health gradients can be generated not only by poverty but also by increasing income inequality [4, 10, 12]. Wang et al. [50] found that the mutual health care system decreases the likelihood of reporting pain/anxiety and improves mobility and self-care among the elderly. Mental health can also be improved by enhancing health knowledge and behaviour and establishing an equitable economic policy [12]. This is also consistent with the findings of one study from Hong Kong, which showed that deprivation of nonmonetary resources caused adverse health outcomes beyond the effect of income poverty [22]. Low health utility scores are also associated with old age, low educational levels, chronic conditions and marital status [21, 22]. Therefore, policies should consider the multidimensionality of poverty.

### Heterogeneous effects

First, the respondents from Central China were mostly affected by poverty in terms of health utility scores and self-care and usual activity. The respondents from western China demonstrated a high probability of reporting problems with pain/discomfort and anxiety/depression, indicating that policy-making should be adjusted based on the local context. Second, residents living longer distances from the nearest healthcare facilities showed increased problems with HRQOL and suffered from limited healthcare service access. Therefore, the availability and quality of healthcare for disadvantaged populations should be continuously improved, including relocation to a habitable place and extended home care services for residents with physical limitations. In respondents with chronic diseases, anxiety/depression was the dimension that was most affected. This result is consistent with the long-term detrimental impact on HRQOL for the population with chronic diseases, especially for mental health among the comorbidity population [51, 52]. Therefore, programs for the poverty-stricken population with chronic diseases should place greater emphasis on psychological health. Third, married subjects and respondents with high educational attainment were associated with high health utility scores [52, 53]. A study in Vietnam showed that women tend to suffer more problems in terms of pain/discomfort and anxiety/depression and have a lower overall HRQOL than men [24]. In this study, male subjects and respondents with high educational levels experienced physical and mental health problems under poverty. Among respondents with different marital statuses, the single population was the least affected in terms of both the health utility scores and the five dimensions. This result may be explained by the high financial strain for the development of the next generation. Married respondents experienced considerable mental health problems, which may be related to high financial constraints for education or marriage costs for their children, especially their sons [20]. Poverty is also related to increased unhealthy behaviour, such as smoking, poor diet and low self-esteem [54]. Hence, active coping strategies should be promoted among these populations [21].

The decline in HRQOL with the advancement of age has been widely acknowledged [52, 55]. In the current study, the middle-aged group was the most vulnerable to poverty, and the elderly suffered more than younger respondents. Since 2009, the rural population aged above 60 can receive at least 55 Chinese Yuan in the New Rural Pension Scheme. A study using the China Family Panel Study data showed that the pension scheme could help to relieve the prevalence of depression symptoms by 25.4% [56]. The decline in quality of life is also induced not only by ageing [57] but also by loneliness and a lack of social participation. The elderly population is resilient to physical health problems [58]. Resilience is linked with longevity and good health status, which aids the elderly population in coping with multidimensional health problems [58]. Therefore, strategies should be more comprehensive, and the mental health of the middle-aged population is important. Policies related to the ageing population must consider both social circumstances and psychological well-being.

This study has several limitations. First, the cross-sectional design could not identify causal associations among the variables of interest. Hence, longitudinal studies should be conducted to confirm the findings. Second, given the limitations of the EQ-5D-3 L instrument, including fewer descriptive capabilities for health status compared with generic instruments, future research using other comprehensive measures of multiple dimensions is needed.

## Conclusions

This study enriches the available studies on the relationship between poverty and health and can inform the government, local healthcare facilities and policy makers. This study also provides a valuable perspective for poverty elimination in other developing countries with similar settings. The results indicate that respondents whose HRQOL was most affected by poverty were those from Central China, male subjects, young population, those with a high educational level and those living far from healthcare facilities. The effect varied among different categories in five dimensions. Considering the vulnerability of health to poverty, we speculated that the population from western China, middle-aged subjects, married respondents, those living far from healthcare facilities and those living with at least one chronic disease suffered the most in terms of mental health. Therefore, mental health should be prioritised for further monitoring and early intervention.

## Data Availability

All research data is available upon reasonable request.

## Abbreviations

EQ-5D-3 L: 3-level EuroQoL 5-dimension instrument
HRQOL: Health-Related Quality of Life
CEM: Coarsened exact matching
OR: Odds Ratio
CI: Confidence interval
DY: Dangyang
SN: Sinan
